# Molecular Targets for 17α-Ethynyl-5-Androstene-3β,7β,17β-Triol, an Anti-Inflammatory Agent Derived from the Human Metabolome

**DOI:** 10.1371/journal.pone.0032147

**Published:** 2012-02-24

**Authors:** Christopher L. Reading, James M. Frincke, Steven K. White

**Affiliations:** 1 Department of Scientific Development, Harbor Biosciences, San Diego, California, United States of America; 2 Department of Medicinal Chemistry, Harbor Biosciences, San Diego, California, United States of America; Shantou University Medical College, China

## Abstract

HE3286, 17α-ethynyl-5-androstene-3β, 7β, 17β-triol, is a novel synthetic compound related to the endogenous sterol 5-androstene-3β, 7β, 17β-triol (β-AET), a metabolite of the abundant adrenal steroid dehydroepiandrosterone (DHEA). HE3286 has shown efficacy in clinical studies in impaired glucose tolerance and type 2 diabetes, and *in vivo* models of types 1 and 2 diabetes, autoimmunity, and inflammation. Proteomic analysis of solid-phase HE3286-bound bead affinity experiments, using extracts from RAW 264.7 mouse macrophage cells, identified 26 binding partners. Network analysis revealed associations of these HE3286 target proteins with nodes in the Kyoto Encyclopedia of Genes and Genomes (KEGG) pathways for type 2 diabetes, insulin, adipokine, and adipocyte signaling. Binding partners included low density lipoprotein receptor-related protein (Lrp1), an endocytic receptor; mitogen activated protein kinases 1 and 3 (Mapk1, Mapk3), protein kinases involved in inflammation signaling pathways; ribosomal protein S6 kinase alpha-3 (Rsp6ka3), an intracellular regulatory protein; sirtuin-2 (Sirt2); and 17β-hydroxysteroid dehydrogenase 1 (Hsd17β4), a sterol metabolizing enzyme.

## Introduction

β-AET is a metabolite of dehydroepiandrosterone (DHEA), the most abundant adrenal sterol in the human metabolome [1]. In rodent studies, the anti-inflammatory activities of exogenous β-AET and DHEA [2] are similar. For β-AET, anti-inflammatory activity in rodent models of multiple sclerosis [3], ulcerative colitis [4], lung inflammation and septic shock [2] has been reported. β-AET also opposes certain glucocorticoid (GC) actions *in vitro* [5] and decreases corticosteroid-induced bone loss in the mouse thermal injury model [6]. Elevated levels of GC are associated with glucose dysregulation and hyperglycemia. Since 11β-HSD1 is involved in the formation of β-AET [7] and GC activity, increased levels of β-AET in obese subjects may relate to the increased levels of this enzyme in obesity [8]. This implies a negative feedback loop, where increased 11β-HSD1 expression results in simultaneous amplification of GC signaling and β-AET formation, which may function to counter undesired aspects of GC signaling and maintain homeostasis. In conditions of GC-associated pathology, this relationship between GC and β-AET may be unbalanced. Furthermore, given that rodents convert DHEA and β-AED into metabolites with significantly more sites of oxidation [9], species differential metabolism may explain why activities observed for these sterols have not translated into human benefit [2], [9].

**Figure 1:**
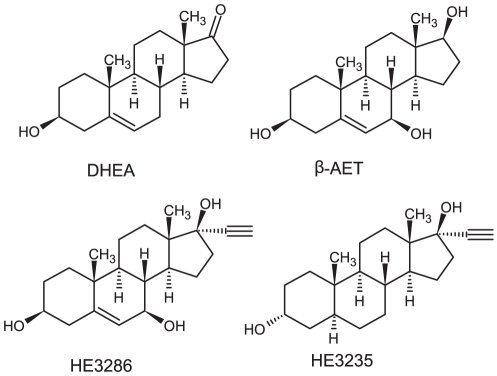
Chemical structures of DHEA, β-AET, HE3286 and HE3235 (negative control).

HE3286, an orally bioavailable synthetic compound (Figure 1), is pharmacologically distinct from androgens, estrogens, corticosteroids, and peroxisome proliferators [10]. HE3286 has demonstrated anti-inflammatory activities similar to β-AET in murine models of ulcerative colitis [4], multiple sclerosis [4], type 1 diabetes mellitus [11], chronic obstructive pulmonary disease [12], and rheumatoid arthritis [4], [13], [14], [15]. All of these models involve attenuation of NF-κB activity and decreased levels of pro-inflammatory cytokines, including TNFα. HE3286 exhibits anti-inflammatory and insulin sensitizing activities in preclinical *db/db* and *ob/ob* mouse, *fa/fa* ZDF rat models of diabetes [10], [16], and in a rare example of species translation for sterols, in contrast to DHEA or β-AET, HE3286 is active in impaired glucose tolerant and type 2 diabetes mellitus human subjects [17].

In mouse macrophages, HE3286 suppressed lipopolysaccharide (LPS)-induced nuclear factor kappa B (NF-κB) reporter gene expression, nuclear localization, and insulin receptor substrate-1 (IRS-1) serine phosphorylation. In addition, this compound decreased activation of the pro-inflammatory kinases IkappaB kinase (IKK), Jun N-terminal kinase (JNK) and p38 mitogen-activated kinase (p38 Mapk). HE3286 also attenuated tumor necrosis factor alpha (TNFα-stimulated inflammation and TNFα-induced adipocyte-stimulated macrophage chemotaxis [10], [16]. HE3286 treatment suppressed levels of the chemokine monocyte chemoattractant protein-1 (MCP-1), along with its cognate receptor, C-C motif chemokine receptor-2, in white adipose tissue [10]. Similarly, in liver and adipose tissue, HE3286 down-regulated inflammatory cytokine/chemokine protein levels and decreased macrophage migration into adipose tissue in Zucker diabetic fatty (ZDF) rats [16]. In obese humans with inflammation-induced diabetes, HE3286 decreased inflammatory cytokine production, C-reactive protein (CRP) and post-prandial glucose levels, leading to a decrease in HbA1c. HE3286 was safe with no drug-related side effects [18]. These properties suggest a potential role for endogenous β-AET, which is lost during inflammation-driven diseases, and exogenous HE3286 in limiting chronic inflammation. Thus, HE3286 represents the first in a new class of anti-inflammatory agents with the potential to treat chronic inflammatory conditions. The clinical results from administering HE3286 prompted the exploration of target proteins in the macrophage, a cell type that is central to the activity of HE3286, using stable isotope labeling with amino acids in culture (SILAC) technology in RAW 264.7 mouse macrophages.

## Results

There were four method variations run in the SILAC experiments. The first and second variations, referred to as forward (A) and reverse (B) conditions, used a solid-phase HE3286-bead compared to non-sterol modified agarose-beads as a control. The third variation, referred to as forward (C) conditions, used solid-phase HE3286-bead in both light and heavy cell preparations where the heavy cell lysate was pre-incubated with 50 µM HE3286 as a soluble competitor. The fourth variation, forward (D) conditions, used solid-phase HE3286-beads with 50 µM HE3235 (NF-κB inactive), pre-incubated in the heavy cells as a soluble competitor and was similarly compared to DMSO buffer using solid-phase HE3286-beads.

The number of proteins found for each experiment and the number identified with a ratio ≥1.5 are delineated in Table 1. A target decoy database of reverse sequences was used to assure a false discovery rate of <1%. This allowed a comparison of different MS database search algorithms for protein identification as there is a known difference in protein identification using different quantification software [19]. We compared search results, found with the Sequest database followed by DTA select processing, with the !Xtandem database, followed by Peptide Prophet and Protein Prophet. In general, the total number of identified proteins was similar. However, while 874 proteins were found in experiment 2A using !Xtandem is extremely close to the 878 discovered initially using Sequest, only 365 coordinate identifications were established. Notably, the alternative !Xtandem methodology, identified 577 proteins with a ratio of >1.5 compared to 372 using the Sequest/DTA analysis. We chose to use the Sequest/DTA algorithms for comparison of separate experiments, as we believed this would provide fewer false positives. Within each experiment we evaluated MS/MS data for each hit to validate the ratios used in our analysis.

**Table 1:** Comparison of the number of proteins identified and the number of protein ratios of >1.5 in representative experiments.

Exp	L/H*	Conditions	#Proteins	#Ratios >1.5
1A	>1	(Heavy+Control Bead)+(Light+3286 Bead)	400	141
1B	<1	(Light+Control Bead)+(Heavy+3286 Bead)	580	162
1C	>1	(Heavy+50 uM HE3286+3286 Bead)+(Light+3286 Bead)	369	8
1D	= 1	(Heavy+50 uM HE3235+3286 Bead)+(Light+3286 Bead)	698	131
2A	>1	(Heavy+Control Bead)+(Light+3286 Bead)	878	372
2B	<1	(Light+Control Bead)+(Heavy+3286 Bead)	919	429
2C	>1	(Heavy+50 uM HE3286+3286 Bead)+(Light+3286 Bead)	942	157

*L/H: (light isotope label/heavy isotope label) expresses the expected outcome ratio for each set of conditions.

Overall, the methodology initially identified 44 candidate binding partners. These were additionally constrained by censoring against a published list of promiscuous binding partners that have been identified through a retrospective analysis of several SILAC experiments [20]. The final analysis revealed 26 HE3286 binding partners that exceeded a 1.5 binding enrichment ratio threshold, listed in Table 2. A description of protein function for these binding partners is included in supplemental Table S1. In theory, the resulting ratio for the 1D experiment should be ≥1.5 in order for this HE3235 control experiment to uncover proteins that non-specifically bind to sterols. However, as HE3235 is similar in chemical structure to HE3286, there may be shared binding targets between these synthetic sterols that can influence this outcome by incorrectly identifying such proteins as non-specific binding partners to HE3286.

**Table 2:** Gene product enrichment ratios in *mus musculus* RAW 264.7 monocyte/macrophage cells using SILAC-based analysis.

Gene ID	IPI number	Gene	Product	A	B	C	D
66885	IPI00880948	Acadsb	acyl-Coenzyme A dehydrogenase	50.9	5.26	1.53	2.57
80911	IPI00857891	Acox3	acyl-Coenzyme A oxidase 3, pristanoyl	46.3	4.17	1.55	3.10
11564	IPI00265471	Adsl	adenylosuccinate lyase	85.3	12.5	2.10	1.90
77559	IPI00662244	Agl	amylo-1,6-glucosidase, 4-alpha-glucanotransferase	23.5	7.70	1.72	1.04
12039	IPI00331555	Bckdha	branched chain ketoacid dehydrogenase E1, alpha	13.7	5.26	1.52	0.98
—	IPI00858053	Cherp	calcium homeostasis endoplasmic reticulum	4.10	14.3	1.91	0.81
234594	IPI00880243	Cnot1	CCR4-NOT transcription complex, subunit 1	30.2	1.79	1.66	0.92
67337	IPI00116747	Cstf1	cleavage stimulation factor, 3′ pre-RNA, subunit 1	45.3	12.5	2.10	0.92
319945	IPI00226787	Flad1	RFad1, flavin adenine dinucleotide synthetase	16.3	12.5	1.66	0.96
51886	IPI00123292	Fubp1	far upstream element binding protein 1	14.2	14.3	2.19	0.43
71665	IPI00387228	Fuca1	fucosidase, alpha-L- 1, tissue	40.5	12.5	2.28	*
11991	IPI00330958	Hnrnpd	heterogeneous nuclear ribonucleoprotein D	4.42	16.7	1.79	0.90
50926	IPI00755892	Hnrpdl	heterogeneous nuclear ribonucleoprotein D-like	24.8	1.92	5.05	0.82
15488	IPI00331628	Hsd17b4	hydroxysteroid (17-beta) dehydrogenase 4	4.97	4.76	1.76	0.95
16971	IPI00119063	Lrp1	low density lipoprotein receptor-related protein 1	9.77	9.09	2.25	0.55
26394	IPI00123518	Lypla2	lysophospholipase 2	2.16	2.50	2.10	0.93
26413	IPI00119663	Mapk1	mitogen-activated protein kinase 1	18.6	6.70	1.94	1.00
26417	IPI00230277	Mapk3	mitogen-activated protein kinase 3	27.8	6.20	1.80	2.13
76890	IPI00126892	Memo1	mediator of cell motility 1	4.63	3.85	1.74	1.08
23983	IPI00128904	Pcbp1	poly(rC) binding protein 1	2.87	12.5	1.62	0.28
231329	IPI00320034	Polr2b	polymerase (RNA) II polypeptide B	12.4	12.5	1.99	0.27
110651	IPI00114333	Rps6ka3	ribosomal protein S6 kinase polypeptide 3	7.57	4.17	1.60	1.02
27054	IPI00317604	Sec23b	Sec23b	11.0	9.09	1.88	0.78
218811	IPI00229483	Sec24c	Sec24 related gene family, member C	6.62	11.1	1.67	0.42
64383	IPI00110265	Sirt2	Sirtuin 2	51.2	8.33	1.80	1.11
19704	IPI00420949	Upf1	UPF1 regulator of nonsense transcripts	19.4	10.0	2.15	0.61

*This protein was not identified in either sample in this experiment.

## Discussion

Specific HE3286 macrophage binding partners were revealed through a combination of solid phase capture and SILAC-based proteomic analysis that resulted in the identification of a set of low abundance proteins including signal transduction-related proteins and transcription factors. The list of specific HE3286 target proteins is more consistent with a biological mechanism operating through a biosystem network paradigm instead of a single target approach. When compared to other strategies for target identification where large lists of proteins are often identified, SILAC affinity experiments produce relatively fewer candidates for inspection. Because the two separate samples are combined before processing and are subjected to an identical experimental protocol, sample background effects are eliminated. Experimental strategies that use soluble competitors significantly reduce the number of potential binding partners; however, multiple binding interactions remain. Functional analyses to establish contributions of potential target proteins to the observed biological phenomena are then required in order to define the most pertinent targets associated with HE3286 biological activities.

**Figure 2:**
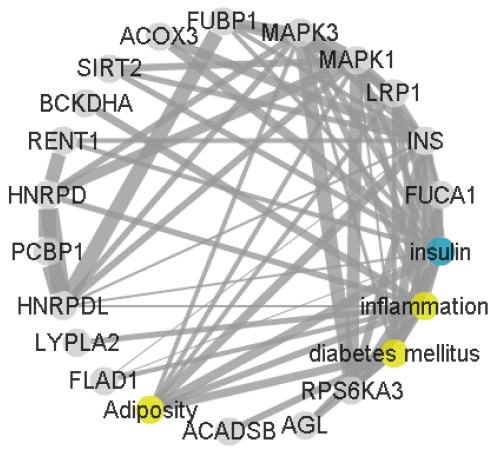
Network of a group of mixed terms using CoPub [**21**] to mine PubMed literature and generate a force directed weighted network where strongly connected terms have thick edges.

Our hypothesis, built on *in vivo* and clinical evidence was that pathways with known association to obesity and insulin resistance; namely insulin signaling, TNFα and LPS signaling and adipose tissue signaling resulting in the release of chemokines, should be effected by our list of associated proteins. Data visualized as a network can be easier to interpret than long lists of proteins, interactions and correlations (Figure 2) [21].

Where once proteins were considered as discrete signals, it is now recognized that many function through complex networks of protein-protein interactions. Using our list of 26 proteins our analysis set increased to 1,569 proteins by including proteins with direct interactions as defined by the BioGRID protein-protein interaction database [22]. KEGG maps for the adipocytokine, toll-like receptor, chemokine and insulin signaling pathways [23] reveal multiple nodes of interaction for these binding partners that are consistent with the observed biological activities of HE3286. The connections of the identified HE3286 binding targets to type 2 diabetes, insulin, adipokine and adipocyte signaling are clear and these KEGG maps are included in a supplemental file Networks S1.

We have subsequently analyzed the data in light of the biological activities and concurrent molecular changes seen with HE3286-treatment protocols. In addition to the anti-inflammatory activities found in preclinical models of autoimmunity and inflammation [4], [11], [12], [13], [14], [15], HE3286 decreases chronic inflammation associated with insulin resistance in rodent models of diabetes [10], [16] and in impaired glucose tolerant and type 2 diabetes mellitus human subjects [17]. HE3286 attenuates NF-κB and TNFα signaling and associated pro-inflammatory cytokines and chemokines, in these settings. Based on these results, we have focused attention on pathways that regulate NF-κB and TNFα stimulation with elaboration of inflammatory cytokines and chemokines. Three particularly important nodes, Lrp1, Mapk1 and Mapk3 were discovered that are consistent with mechanistic explanations for HE3286 anti-inflammatory and insulin-sensitizing activities.

Lrp1 was identified as the only surface receptor that binds to HE3286 on RAW264.7 cells. Lrp1 is regulated by insulin signaling [24], [25] and is important in lipid clearance and glucose tolerance. Additionally, Lrp1 signaling results in anti-inflammatory effects. Mice with an adipocyte-specific inactivation of Lrp1 displayed delayed postprandial lipid clearance, reduced body weight, smaller fat stores, improved glucose tolerance, and resistance to dietary fat-induced obesity and glucose intolerance [26]. Inactivation of the Lrp1 intracellular NpxYxxL motif enhances post prandial dyslipidemia and atherosclerosis, and increases proapoptotic effects *via* increased secretion of TNFα in macrophages [27].

Lrp1 is one regulator of the tumor necrosis factor receptor-1 (TNFR1) and the IKK-NF-κB pathway. Lrp1 deficient macrophages have decreased NF-κB signaling and MCP-1 expression, due to down-regulation of TNFR1 and inhibition of autocrine TNFR1-initiated cell signaling [28]. Lrp1 binds Apolipoprotien E (ApoE) [29], and ApoE induces the anti-inflammatory M2 phenotype in macrophages [30]. Lrp1 has also been shown to interact with scaffolding and signaling proteins *via* its intracellular domain in a phosphorylation-dependent manner [31].

In addition to a cell-surface interaction with Lrp1, HE3286 also binds Mapk1 and 3, and this binding likely modulates scaffold interactions, which allows cross-talk between Ras-Erk and Lrp1 signaling. Mapk1 and Mapk3 (Erk2, Erk1 respectively) are integral mediators of inflammatory signal transduction that are central to obesity. Mice deficient in a natural Erk inhibitor, p62, have a high level of Erk activity and develop mature-onset obesity and insulin resistance [32]. Knockout of Erk1 in these mice decreases their adiposity and insulin resistance [33]. Activation of TLR2 and TLR4 with fatty acids [34], [35] contributes to NF-κB activation, increased inflammation, and insulin resistance. Tpl2 kinase is up-regulated in adipose tissue in obese mice and human subjects and is reported to mediate NF-κB and TNFα effects upon Erk activation [36]. Inhibitors of IKKβ or TPL2 eliminated TNFα but not insulin-mediated Erk1 and Erk2 activation and also eliminated IRS-1 Ser636 phosphorylation stimulated by TNFα in 3T3-L1 adipocytes.

We have shown that addition of HE3286 *in vitro* to Mapk1 in the presence of activated Mek1 does not affect phosphorylation activity of Mapk1 nor does its addition to phosphorylated Mapk1 effect phosphorylation of a peptide substrate (Reading, unpublished observations). This finding suggests that HE3286 binding perturbs scaffolding of Mapk within protein complexes to attenuate Ras-Erk signal transduction rather than by acting through direct inhibition of the kinase's catalytic domain. This may be important to the safety of this molecular class as direct inhibition of kinase catalytic activity by ATP site-dependent inhibitors often leads to undesirable toxicities. The development of “soft drugs” that attenuate signaling has been suggested as an alternative to high affinity molecular entities that neutralize protein action.

While the Lrp1, Mapk1, and Mapk3 are arguably central to the biological phenomena observed for HE3286, other identified binding partners that can also significantly contribute to these effects are enzymes central to glucose metabolism (Agl, glycogen degradation) and fatty acid metabolism (Acadsd, short/branched chain acyl-CoA dehydrogenase: Acox3, acyl-CoA oxidase 3; Hsd17b4, fatty acid β-oxidase; Lypla2, lysophospholipase). Sirt2, a protein of recent interest for its role in intracellular regulation through a mono-ADP-ribosyltransferase activity was also identified as an HE3286 target protein. The steroidogenic enzyme Hsd17b4 oxidation of the C-17 hydroxyl function of C-19 sterols is known to modify the action of 17-hydroxysterols. In a pro-inflammatory environment, oxidation of β-AET deactivates its anti-inflammatory properties thereby permitting the cell to execute its inflammation program. HE3286 a chemically modified version of β-AET, should inhibit the action of this hydroxysteroid dehydrogenase to exert an anti-inflammatory effect.

The SILAC experiments generated a list of 26 target proteins specific to HE3286. This result of multiple target proteins is consistent with our expectations given the diverse biological activities for HE3286 that have been observed in separate *in vitro* and *in vivo* models and the involvement of the identified binding partners to these activities. Future additional functional activity assays will serve to detail the contributions of each binding partner to the broad anti-inflammatory, insulin-sensitizing, and lipid regulating activities of HE3286. Experiments with Lrp1 macrophage knockout mice should further establish the role of HE3286 acting upon this binding partner.

## Materials and Methods

### Materials

All chemicals and solvents, including, **l**-arginine-^13^C_6_^15^N_4_-HCl, **l**-lysine-^13^C_6_^15^N_2_-HCl, 4-iodobenzylamine hydrochloride, 9-fluorenylmethyloxycarbonyl chloride (FMOC-Cl), triethylamine, ethanolamine, palladium diacetate, triphenyl phosphine, cuprous iodide, piperidine, urea, iodoacetamide, ammonium bicarbonate, Tris(2-carboxyethyl)phosphine-HCl (TCEP), sodium chloride, formic acid, THF (anhydrous), DMF (anhydrous), and acetonitrile (ACN, HPLC grade) were purchased from Sigma-Aldrich (St. Louis, MO).

N-hydroxy-succinimide-activated agarose slurry (cat#26200) and SILAC RPMI media (cat#89984) were purchased from Thermo Scientific (Rockfield, IL). HEPES buffer was purchased from Mediatech (Manassas, VA) and the dialyzed fetal bovine serum (FBS) was purchased from Sigma-Aldrich (St. Louis, MO).

### Affinity capture beads

The preparation of an amino analog of HE3286 for linkage to NHS-activated agarose beads was completed using a Sonogashira palladium cross-coupling reaction between the C-17 alkynyl group and a FMOC protected 4-iodobenzylamine [37]. The FMOC protecting group was removed following the cross-coupling by heating in piperidine. The HE3286-benzyl amine was coupled to the NHS-activated bead according to standard protocol [38]. For evaluation of non-specific binding of proteins onto the affinity sorbent, we also prepared control agarose beads that were subjected to the same treatments as the HE3286-agarose beads, except the HE3286-amine was replaced with ethanolamine.

### Protein capture

The mouse macrophage cell line RAW 264.7 (ATCC CRL-2278) cells were grown for at least 6 cell divisions in media supplemented with 5% dialyzed FBS, in a humidified air atmosphere with 5% CO_2_. All standard SILAC media preparation and labeling steps were followed as previously described [39]. Separate RAW 264.7 cell cultures, SILAC labeled with either light or heavy amino acids, were lysed in ice-chilled Tris buffer (50 mM, pH 8) that included protease inhibitors. Light and heavy lysate protein concentrations were estimated using the DC Protein Assay (Bio-Rad, Hercules CA). Affinity enrichment mixtures were incubated overnight (4°C, 16 h) on an end-over-end rotator, and beads were combined for work-up following necessary individual washing steps.

Proteins enriched in the SILAC affinity pull-down experiments were reduced and alkylated on bead, in 5 mM TCEP and 10 mM iodoacetamide, respectively. Trypsin was then added and the vials were incubated at 37°C overnight on a thermomixer.

### Protein analysis by LC-MS

The LC/MS analysis followed a standard protocol for the MuDPIT technique described in the referenced protocol [40]. There were 5 salt gradients flushed through the capillary column prior to a gradient HPLC method that eluted peptides fractions into the Mass Spec for MS and MS/MS spectra collection. Tandem mass spectra were searched using the Sequest algorithm (v 3.0) against the mouse database (ipi.MOUSEv368.fasta) from the European Bioinformatics Institute (EBI).

### SILAC study design

**Figure 3:**
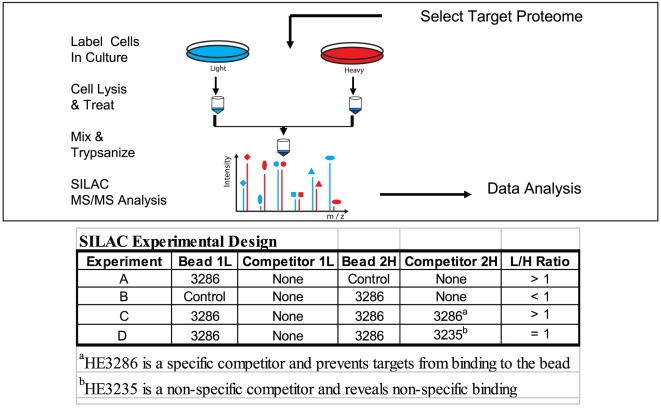
The experimental strategy for identification of proteins that bind HE3286 in the RAW 264.7 mouse macrophage proteome. Cells are grown in light (natural isotope) growth medium or heavy medium, containing amino acids enriched in stable isotopes (^13^C and ^15^N). Both the heavy and light lysates were incubated with small molecule loaded beads or beads without drug (control) to enrich proteins using the drug as an affinity probe. Alternatively, the addition of a soluble form of the drug is added to compete with the affinity matrix for binding to target proteins. Proteins bound to the affinity matrix are digested on bead to peptides and quantified by LC-MS/MS. Target proteins are identified by analyzing the resulting abundance ratios *XIC*_light_/*XIC*_heavy_.

The experimental design used to identify HE2386 binding partners in the RAW 264.7 cell proteome (Figure 3) was adapted to this application from a previously described method [41]. For this purpose, HE3286 was coupled by an amine substituted linker to a solid phase agarose bead to retain proteins that could be classified as candidate binding partners (i.e., HE3286 target proteins). Most proteins non-specific to HE3286 were washed off the beads and candidate binding partners that remained adsorbed to the solid phase were subsequently released and captured for identification. This resulting mixture will contain direct target proteins to HE3286 or alternatively secondary accessory binders (due to protein-protein interactions) such as those involved in scaffold assembly of protein complexes. Control beads were used in each experiment to identify non-specific absorption by the agarose bead, and these non-specific absorbed proteins were removed from the candidate list by subtracting the two data sets. Thorough mixing of the solid phase with microliter quantities of the reagents and cell extracts was an important element to reduce the variability in each experiment. In another experiment, soluble HE3286 was used as a competitor to inhibit bead binding of candidate target proteins. Subtracting the soluble competitor data set from a buffer control produced a decreased ratio (>1.5) for HE2386-specific binding partners in comparison to the previous list of candidate binding partners and also identified non-specific binding proteins. The soluble competitor experiment is sensitive to the presence of residual competitor during workup.

In a separate approach for identifying non-specific binding proteins, the structurally similar sterol 17 α -ethynyl -5α-androstane-3α, 17β-diol (HE3235), which was inactive in our *in vitro* biochemical assay for NF-κB inhibition, was used as a non-specific soluble competitor. This eliminated proteins that bound to the solid phase with low affinity, which are presumed to be non-specific in nature and unrelated to the observed biological activity for HE3286. Importantly, HE3235 in the soluble competitor experiment may compete with specific HE3286-bound target proteins if binding sites for their respective binding partners are structurally similar. This complication may eliminate a participating target protein and produce a false negative result. Variability inherent in the SILAC method is resolved through experimental repetition.

## Supporting Information

Table S1 Brief description of the 26 gene products bound by HE3286.
(XLS)

Networks S1 Interactions from the BioGRID protein-protein interaction database (v. 3.1.74) suggest 1,569 possible proteins under the influence of HE3286. Using this expanded set of proteins, multiple nodes within pathways found in the KEGG database suggest that HE3286 may be involved in various pathways linked to inflammation. In the included three example pathways, Adipocytokine Signaling, Insulin Signaling, and Type II Diabetes Mellitus, nodes are highlighted in green for proteins found in this SILAC study and nodes are highlighted in red if the targets are involved in a protein-protein interaction. The two column tables below each pathway list the HE3286 bound proteins in the first column (black) while the partnered protein (listed in BioGRID) is shown in the second column, highlighted in the same color as in the pathway above.
(PDF)
