# Supplementary material for: Molecular Targets for 17α-Ethynyl-5-Androstene-3β,7β,17β-Triol, an Anti-Inflammatory Agent Derived from the Human Metabolome
Source: PLoS One. 2012 Feb 24;7(2):e32147. doi: 10.1371/journal.pone.0032147 (PMC3286445; doi:10.1371/journal.pone.0032147)
Supplement: Networks S1 — Interactions from the BioGRID protein-protein interaction database (v. 3.1.74) suggest 1,569 possible proteins under the influence of HE3286. Using this expanded set of proteins, multiple nodes within pathways found in the KEGG database suggest that HE3286 may be involved in various pathways linked to inflammation. In the included three example pathways, Adipocytokine Signaling, Insulin Signaling, and Type II Diabetes Mellitus, nodes are highlighted in green for proteins found in this SILAC study and nodes are highlighted in red if the targets are involved in a protein-protein interaction. The two column tables below each pathway list the HE3286 bound proteins in the first column (black) while the partnered protein (listed in BioGRID) is shown in the second column, highlighted in the same color as in the pathway above. (PDF) [file pone.0032147.s002.pdf]

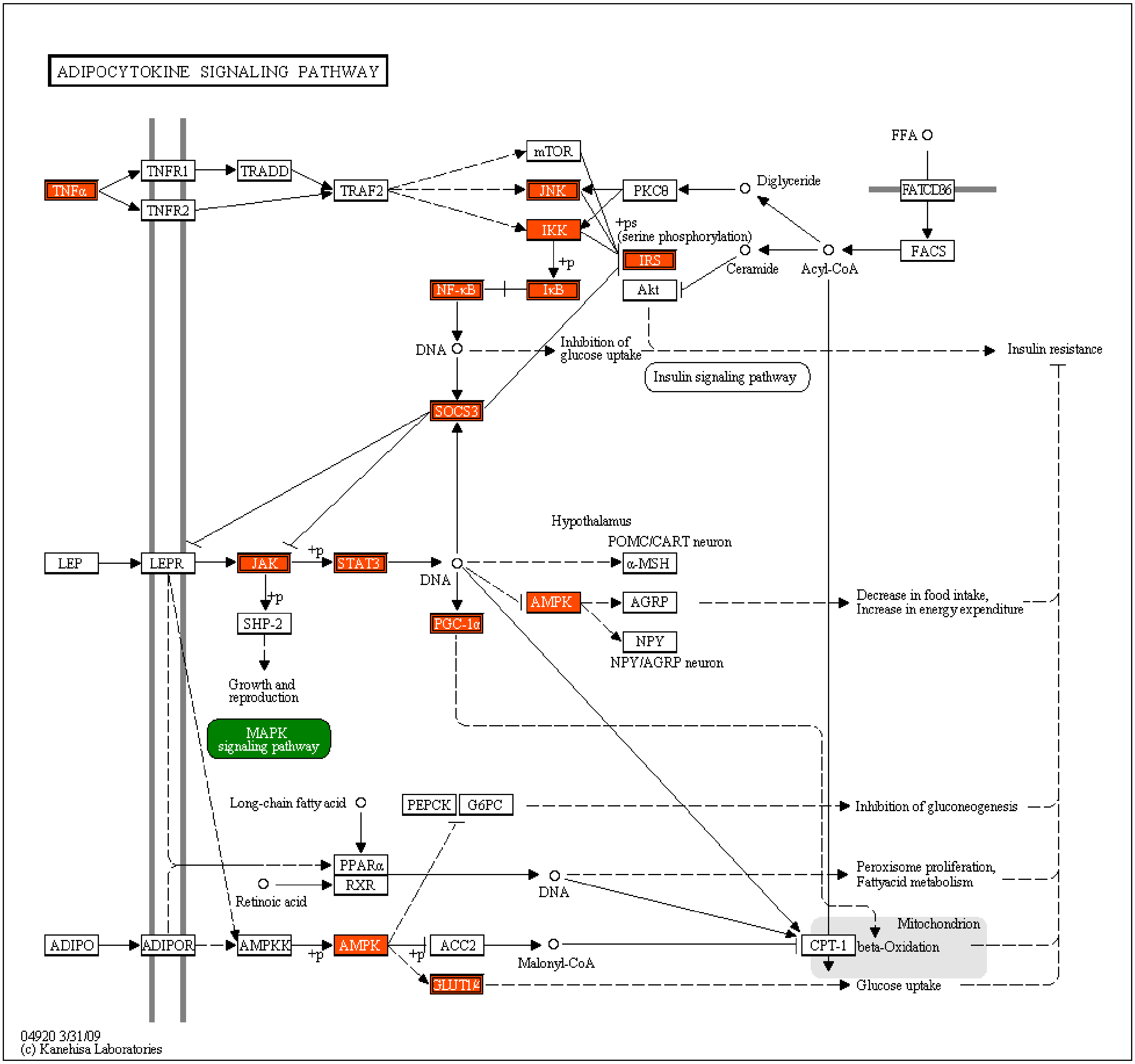

Fubp1	MAPK8
Lrp1	SLC2A1
Mapk1	IKBKB
Mapk1	IRS1
Mapk1	IRS2
Mapk1	IRS4
Mapk1	MAPK1
Mapk1	MAPK3

Mapk1	MAPK8
Mapk1	MAPK9
Mapk1	PPARGC1A
Mapk1	SOCS1
Mapk1	SOCS2
Mapk1	SOCS3
Mapk1	SOCS6
Mapk1	TNF

Mapk3	IRS1
Mapk3	IRS2
Mapk3	IRS4
Mapk3	MAPK1
Mapk3	MAPK3
Mapk3	MAPK10
Mapk3	MAPK8
Mapk3	MAPK9

Mapk3	SOCS1
Mapk3	SOCS2
Mapk3	SOCS3
Mapk3	SOCS6
Mapk3	TNF
Sirt2	PRKAB1

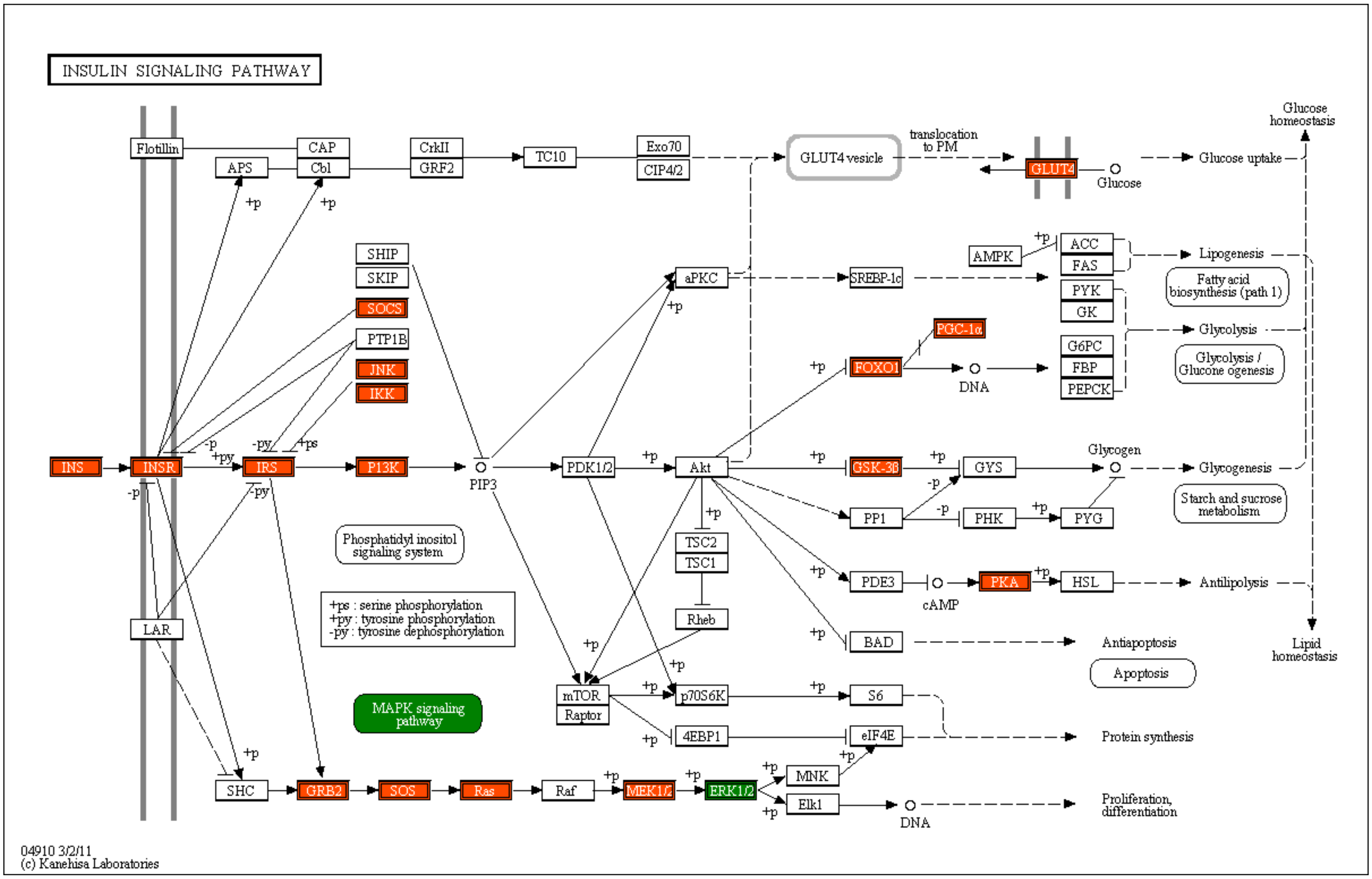

Fubp1	MAP2K1
Fubp1	MAPK1
Fubp1	MAPK3
Fubp1	MAPK8
Mapk1	IKBKB
Mapk1	INS
Mapk1	INSR
Mapk1	IRS1
Mapk1	IRS2
Mapk1	IRS4
Mapk1	MAP2K1
Mapk1	MAP2K2
Mapk1	MAPK1
Mapk1	MAPK3

Mapk1	MAPK8
Mapk1	MAPK9
Mapk1	PIK3R1
Mapk1	PIK3R3
Mapk3	INS
Mapk3	INSR
Mapk3	IRS1
Mapk3	IRS2
Mapk3	IRS4
Mapk3	MAP2K1
Mapk3	MAP2K2
Mapk3	MAPK1
Mapk3	MAPK10
Mapk3	MAPK3

Mapk3	MAPK8
Mapk3	MAPK9
Mapk3	PIK3CG
Mapk3	PIK3R1
Mapk3	PIK3R3
Rps6ka3	INS
Rps6ka3	MAP2K1
Rps6ka3	MAP2K2
Rps6ka3	MAPK1
Rps6ka3	MAPK3
Sirt2	PRKAB1

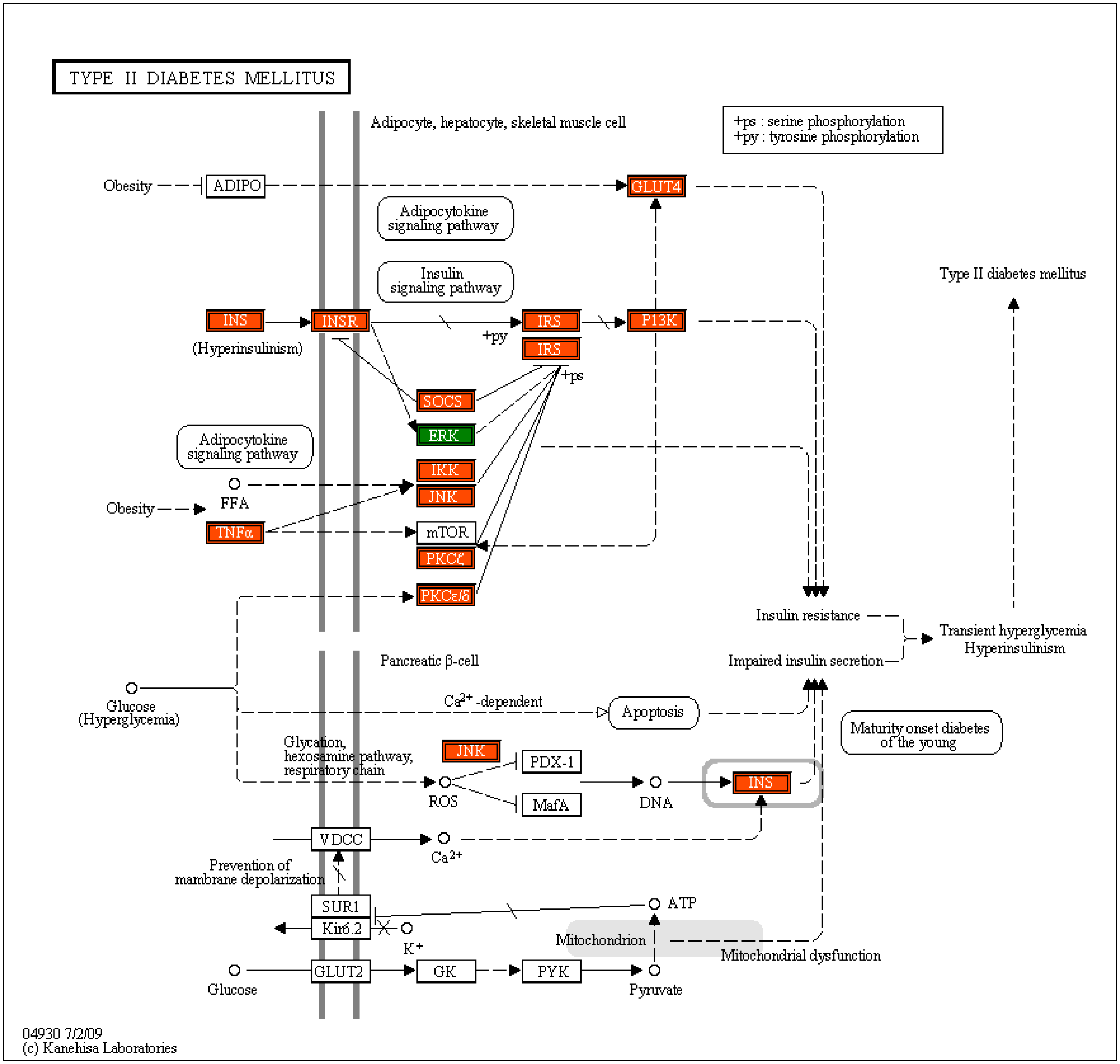

Fubp1	GLUT4
Fubp1	MAPK1
Fubp1	MAPK3
Fubp1	MAPK8
Lrp1	GLUT4
Mapk1	IKBKB
Mapk1	INS
Mapk1	INSR
Mapk1	IRS1
Mapk1	IRS2
Mapk1	IRS4
Mapk1	MAPK1
Mapk1	MAPK3

Mapk1	MAPK8
Mapk1	MAPK9
Mapk1	PIK3R1
Mapk1	PIK3R3
Mapk1	PRKCD
Mapk1	PRKCE
Mapk1	PRKCZ
Mapk1	SOCS1
Mapk1	SOCS2
Mapk1	SOCS3
Mapk1	TNF
Mapk3	INS
Mapk3	INSR

Mapk3	IRS1
Mapk3	IRS2
Mapk3	IRS4
Mapk3	MAPK1
Mapk3	MAPK10
Mapk3	MAPK3
Mapk3	MAPK8
Mapk3	MAPK9
Mapk3	PIK3CG
Mapk3	PIK3R1
Mapk3	PIK3R3
Mapk3	PRKCD
Mapk3	PRKCZ

Mapk3	SOCS1
Mapk3	SOCS2
Mapk3	SOCS3
Mapk3	TNF
Rps6ka3	INS
Rps6ka3	MAPK1
Rps6ka3	MAPK3
Rps6ka3	PRKCD
Rps6ka3	PRKCE
Rps6ka3	PRKCZ
